# Comparison of trunk muscle exercises in supine position during short arm centrifugation with 1 g at centre of mass and upright in 1 g

**DOI:** 10.3389/fphys.2022.955312

**Published:** 2022-08-17

**Authors:** Timo Frett, Leopold Lecheler, Martin Speer, David Marcos, Dominik Pesta, Uwe Tegtbur, Marie-Therese Schmitz, Jens Jordan, David Andrew Green

**Affiliations:** ^1^ German Aerospace Center, Institute of Aerospace Medicine, Cologne, Germany; ^2^ European Space Agency, Cologne, Germany; ^3^ Universidad Autónoma Madrid, Madrid, Spain; ^4^ Center for Endocrinology, Diabetes and Preventive Medicine (CEDP), University Hospital Cologne, Cologne, Germany; ^5^ Cologne Excellence Cluster on Cellular Stress Responses in Aging-Associated Diseases (CECAD), Cologne, Germany; ^6^ Hannover Medical School, Institutes of Sports Medicine, Hannover, Germany; ^7^ Informatics and Epidemiology, Institute of Medical Biometry, Medical Faculty, University of Bonn, Bonn, Germany; ^8^ Chair of Aerospace Medicine, University of Cologne, Cologne, Germany; ^9^ King’s College London, London, United Kingdom; ^10^ Space Medicine Team, European Astronaut Centre, European Space Agency, Cologne, Germany; ^11^ KBRwyle GmbH, Cologne, Germany

**Keywords:** artificial gravity, exercise, countermeasure, spaceflight, trunk muscle atrophy

## Abstract

Spaceflight is associated with reduced antigravitational muscle activity, which results in trunk muscle atrophy and may contribute to post-flight postural and spinal instability. Exercise in artificial gravity (AG) performed *via* short-arm human centrifugation (SAHC) is a promising multi-organ countermeasure, especially to mitigate microgravity-induced postural muscle atrophy. Here, we compared trunk muscular activity (mm. rectus abdominis, ext. obliques and multifidi), cardiovascular response and tolerability of trunk muscle exercises performed during centrifugation with 1 g at individual center of mass on a SAHC against standard upright exercising. We recorded heart rate, blood pressure, surface trunk muscle activity, motion sickness and rating of perceived exertion (BORG) of 12 participants (8 male/4 female, 34 ± 7 years, 178.4 ± 8.2 cm, 72.1 ± 9.6 kg). Heart rate was significantly increased (*p* < 0.001) during exercises without differences in conditions. Systolic blood pressure was higher (*p* < 0.001) during centrifugation with a delayed rise during exercises in upright condition. Diastolic blood pressure was lower in upright (*p* = 0.018) compared to counter-clockwise but not to clockwise centrifugation. Target muscle activation were comparable between conditions, although activity of multifidi was lower (clockwise: *p* = 0.003, counter-clockwise: *p* < 0.001) and rectus abdominis were higher (clockwise: *p* = 0.0023, counter-clockwise: < 0.001) during centrifugation in one exercise type. No sessions were terminated, BORG scoring reflected a relevant training intensity and no significant increase in motion sickness was reported during centrifugation. Thus, exercising trunk muscles during centrifugation generates comparable targeted muscular and heart rate response and appears to be well tolerated. Differences in blood pressure were relatively minor and not indicative of haemodynamic challenge. SAHC-based muscle training is a candidate to reduce microgravity-induced inter-vertebral disc pathology and trunk muscle atrophy. However, further optimization is required prior to performance of a training study for individuals with trunk muscle atrophy/dysfunction.

## Introduction

Long term exposure to microgravity (μg) is associated with multi-organ deconditioning, including muscle atrophy (Fitts et al., 2010; Gopalakrishnan et al., 2010), reduced bone mineral density (LeBlanc et al., 2000; Lang et al., 2017), neurovestibular dysfunction (zu Eulenburg et al., 2021), and cardiovascular deconditioning (Gallo et al., 2020; Patel, 2020). Moreover, chronic cephalic fluid redistribution promotes blood volume loss, and may contribute to ocular and cerebral changes, known as spaceflight-associated neuro-ocular syndrome (SANS) (Clément and Bukley, 2007; Roberts et al., 2017; Lee et al., 2018). As opposed to trunk muscle loss, skeletal muscle atrophy is more pronounced in the lower body due to the reduction in locomotion and postural activation (Fitts et al., 2001; LeBlanc et al., 2007; Trappe et al., 2009; Fitts et al., 2010; Tanaka et al., 2017; Oliva-Lozano and Muyor, 2020). Nevertheless, trunk muscle atrophy occurs despite daily integrated resistance and aerobic countermeasures on the International Space Station (ISS) (Petersen et al., 2016). In fact, in-flight countermeasures do not replicate the mechanical loading associated with equivalent exercise on earth (Kozlovskaya and Grigoriev, 2004; Smith et al., 2008; Guinet et al., 2009; Gopalakrishnan et al., 2010; Lee et al., 2015). For instance, ISS treadmill running with a harness provides up to 80% axial loading, but results in only 25–46% peak ground reaction forces compared to terrestrial conditions (Cavanagh et al., 2010). Thus, exercises seem to be less effective when performed in weightlessness. Trunk muscle atrophy and reduced muscle tone may contribute to inter-vertebral disc (IVD) pathology, including disk desiccation and osteophytes (Garcia et al., 2018), and contributes to an apparent increased risk of IVD herniation post-flight (Johnston et al., 2010; McNamara et al., 2019). Current in-flight resistance training using the Advanced Resistive Exercise Device (ARED) appears to have attenuated bone mineral density loss (Sibonga et al., 2019) but fails to entirely mitigate musculoskeletal deconditioning (Lang et al., 2017). While ARED exercise is insufficient to activate trunk musculature, it may result in high instantaneous axial loading and–in combination with an unloaded spinal column - may thus contribute to post-flight IVD pathologies (Green and Scott, 2017). Post-flight rehabilitation seeks to progressively activate trunk musculature including lumbar multifidus and transversus abdominis muscles in order to promote functional postural and spinal stability (Hides et al., 2016).

To ensure physical performance of crewmember, more effective, efficient and safe multi-organ countermeasures–that include functional activation of trunk muscles - are required for future deep-space exploration missions (Scott et al., 2019). The generation of Artificial Gravity (AG) *via* short-arm human centrifugation (SAHC) is a promising approach to ameliorate multi-organ de-conditioning, including spinal dysfunction (Clément et al., 2004). During centrifugal acceleration, SAHC generates the sensation of ‘standing-up’ despite supine position with the feet placed against a footplate. The ground reaction forces at the feet are proportional to the gravitational (g) level at that point which increases with distance from the axis of rotation (Clément et al., 2004). As a result, postural-related musculoskeletal loading and muscle activation can be induced (GJNm, 2017). However, unlike long-arm centrifugation, SAHC also generates a hydrostatic pressure gradient towards the feet, which can present a significant orthostatic challenge (Clément et al., 2016; Laing et al., 2020).

A number of short-duration (5–14 days) head-down bed rest studies–a common ground-based analogue of μg (Hargens and Vico, 2016) - suggest that repeated passive (i.e. no movement or isometric ‘standing’ during rotation) artificial gravity exposure may be protective against bed rest-induced musculoskeletal deconditioning (Rittweger et al., 2015) and orthostatic intolerance (Stenger et al., 2012). Furthermore, we demonstrated that daily passive centrifugation at 1 *g* at the participants Center of Mass (CoM) is well tolerated during a 60-days bed rest period (Frett et al., 2020a). Nevertheless, 30 min of daily passive centrifugation with 1 g at the CoM provides a relatively low physiological load (Kramer et al., 2020a), and recent data suggests that it is insufficient to ameliorate bed rest-induced multi-organ deconditioning (Attias et al., 2020; Hoffmann et al., 2021).

Early work with AG higher than 1 g suggested that movement during rotation precipitates disorientation and/or motion sickness (Bertolini and Straumann, 2016) due to cross-coupled angular acceleration and induction of Coriolis forces (Bertolini and Straumann, 2016), in addition to orthostatic intolerance (Goswami et al., 2015). In case the g load is moderate (e. g. 1 g at CoM) and the exercise-related head and body motion is congruent, moderate movement appears well tolerated (Frett et al., 2020b). Indeed, plyometric exercises such as jumping can be performed during SAHC, albeit requiring familiarization to generate reaction forces equivalent to ground conditions (Frett et al., 2020b; Kramer et al., 2020b; Dreiner et al., 2020). Furthermore, intense cycle training, knee bends, or heel raises were partially effective to preserve orthostatic tolerance, exercise capacity as well as thigh muscle volume, knee extensor and plantar flexor performance during 4–21 days bed rest (Akima et al., 2005; Iwase, 2005; Caiozzo et al., 2009; Stenger et al., 2012; Li et al., 2017). Cycle ergometry at 40–60 W with 1.2 *g* at heart level (3.5 *g* at feet) ameliorated plasma volume, orthostatic tolerance time, and VO2max during short duration (4–14 days) bed rest (Iwasaki et al., 2005; Yang et al., 2011).

Isometric abdominal, lateral stabilization, or trunk rotation and isometric abdominal exercises are promising approaches to promote trunk stabilization and muscle activation and thus potentially ameliorate μg-induced muscle atrophy and subsequent spinal column dysfunction. However, such exercises need to be performed with an appropriate gravitational (axial) load. Thus, physiological responses during trunk muscle exercising performed on a SAHC as part of a multidimensional countermeasure approach need to be investigated. Whether or not trunk muscle exercises during AG are tolerated and induce comparable trunk muscle activation during standard upright exercising is unknown. As rotational direction could lead to one-sided strain (Kramer et al., 2020b), we compared both centrifuge directions (clockwise and counter-clockwise) in randomized order. We hypothesize that trunk and back muscle exercises will be tolerable due to a reduced amount of required head movements.

The aim of this study was therefore to compare trunk muscular activity (mm. rectus abdominis, ext. obliques and multifidi), cardiovascular response and tolerability of trunk muscle exercises performed during clockwise and counter-clockwise SAHC with 1 g at the individual CoM compared to that generated in an upright (1 g) position.

## Methods

Twelve recreationally active (least twice weekly running and/or functional training) individuals (8 men/4 females, age 34 ± 7 years, height 178.4 ± 8.2 cm, weight 72.1 ± 9.6 kg) provided written informed consent for participation in this study which was approved by the North Rhine ethical committee (Number: 6000223393) and prospectively registered in the German Clinical Trial Register (DRKS: S00021750).

All participants completed a brief medical questionnaire and underwent a standardized centrifuge medical screening including clinical-chemical analyses of blood and urine, stress electrocardiogram, and orthostatic testing. Participants were excluded if they had acute pain or any significant current or past musculoskeletal, cardiovascular or neurological disorder or injury that could affect the ability to perform exercise. No anti-emetic medication was allowed prior to testing and light food (cereal bars) and non-sparkling water were provided to ensure adequate hydration and glycemia.

**FIGURE 1:**
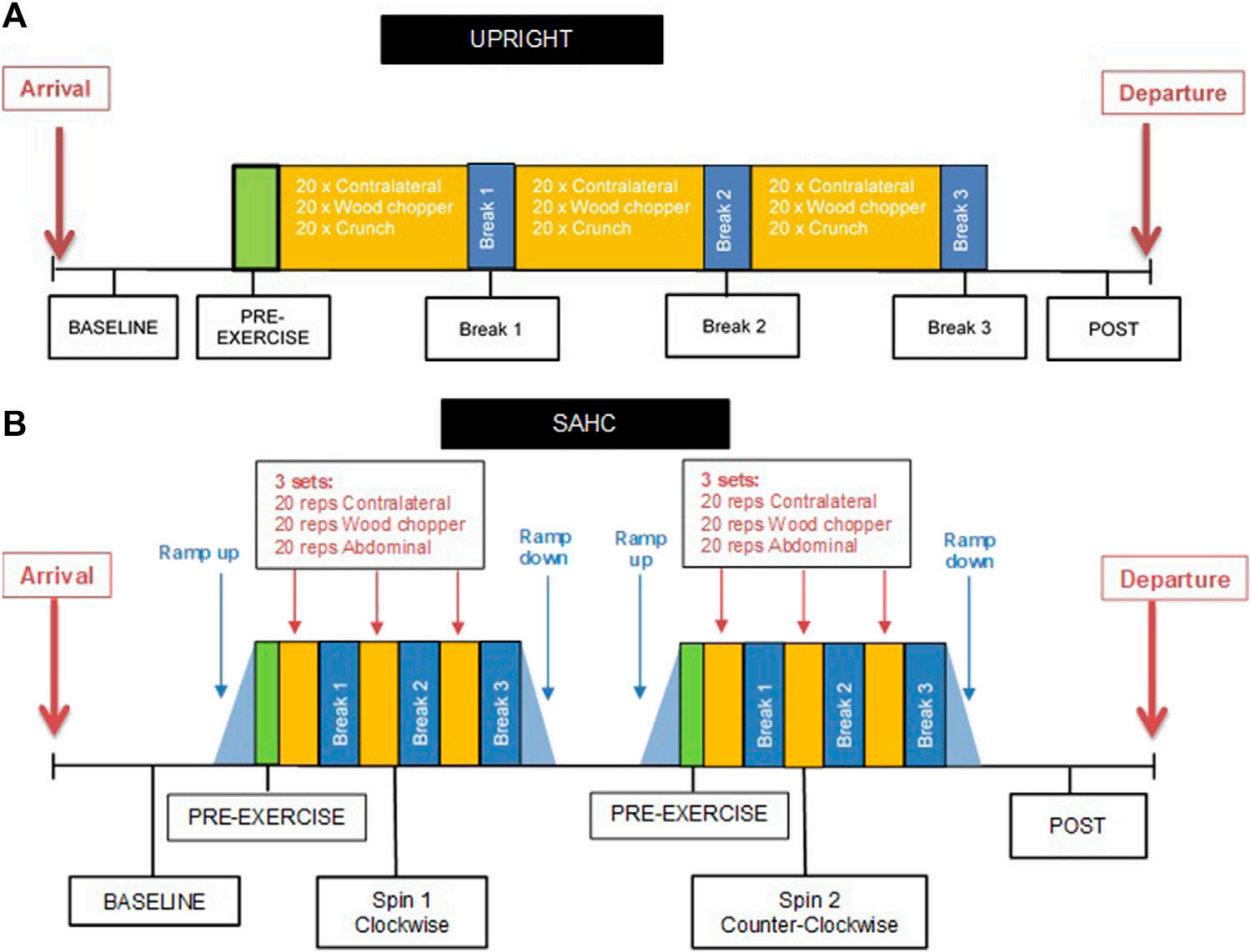
**(A)** Schematic of the UPRIGHT protocol prior to supine trunk muscle exercise (BASELINE), when standing upright (PRE-EXERCISE), between exercise sets (Break 1, Break 2, Break 3) and after exercising (POST). **(B)** Schematic of the short-arm human centrifugation (SAHC) protocol (CLOCKWISE and COUNTER-CLOCKWISE in random order) prior to supine trunk muscle exercise (BASELINE), on the SAHC with 1 g at the participant’s center of mass (PRE-EXERCISE), between exercise sets (Break 1, Break 2, Break 3) and after exercising (POST).

Participants attended the laboratories at:envihab (DLR, Cologne, Germany) on two occasions separated by at least three resting days. Participants were familiarized with the equipment, testing procedures and exercises prior to the first session. Each session included resting measurements in supine position (BASELINE), immediately prior to exercises (PRE-EXERCISE) and after exercise (POST). Participants performed three sets of trunk/upper body exercises in a randomized order: lateral stabilization (Contralateral), abdominal rotation (Wood Chopper) and abdominal isometric (Crunch), each separated by short (30s) periods of rest (BREAK 1, BREAK 2, BREAK 3). All exercises were: either performed standing upright (UPRIGHT), or supine on the SAHC at an angular velocity sufficient to generate 1 g at that individual’s CoM (ratio center of mass to body height 56% for male/54% for female) in a randomized order (Figure 1A).

The centrifuge session consisted of 2 separate centrifuge runs, one in the clockwise (CLOCKWISE), and the other in counter-clockwise (COUNTER-CLOCKWISE) direction, also in a randomized order (Figure 1B). During the ramp up/down phases (de)acceleration did not exceed 5°s^−2^ to minimize the risk of vestibular-induced tumbling sensations*.* On the centrifuge, participants were secured in a supine position on a horizontal sledge system against a fixed footplate and were instructed to avoid unnecessary head movements during centrifugation to minimize the provocation of disorientation/motion sickness symptoms.

For each exercise session, participants performed 20 repetitions of Contralateral, 10 repetitions per side for Wood Cho pper (20 in total), and 20 repetitions of Crunch exercise. The target muscle groups for contralateral exercise are the lateral abdominal (external obliques) and back (multifidi) muscles, whereas for the wood chopper it is the lateral abdominal muscle and for crunch exercises it is the medial abdominal (upper and lower rectus abdominis) muscle. Recorded audio start/stop and pacing instructions were provided for each exercise with participants having balance air pillows (Sissel, Bad Dürkheim Germany) placed under the feet to promote dynamic hip stabilization.

**FIGURE 2:**
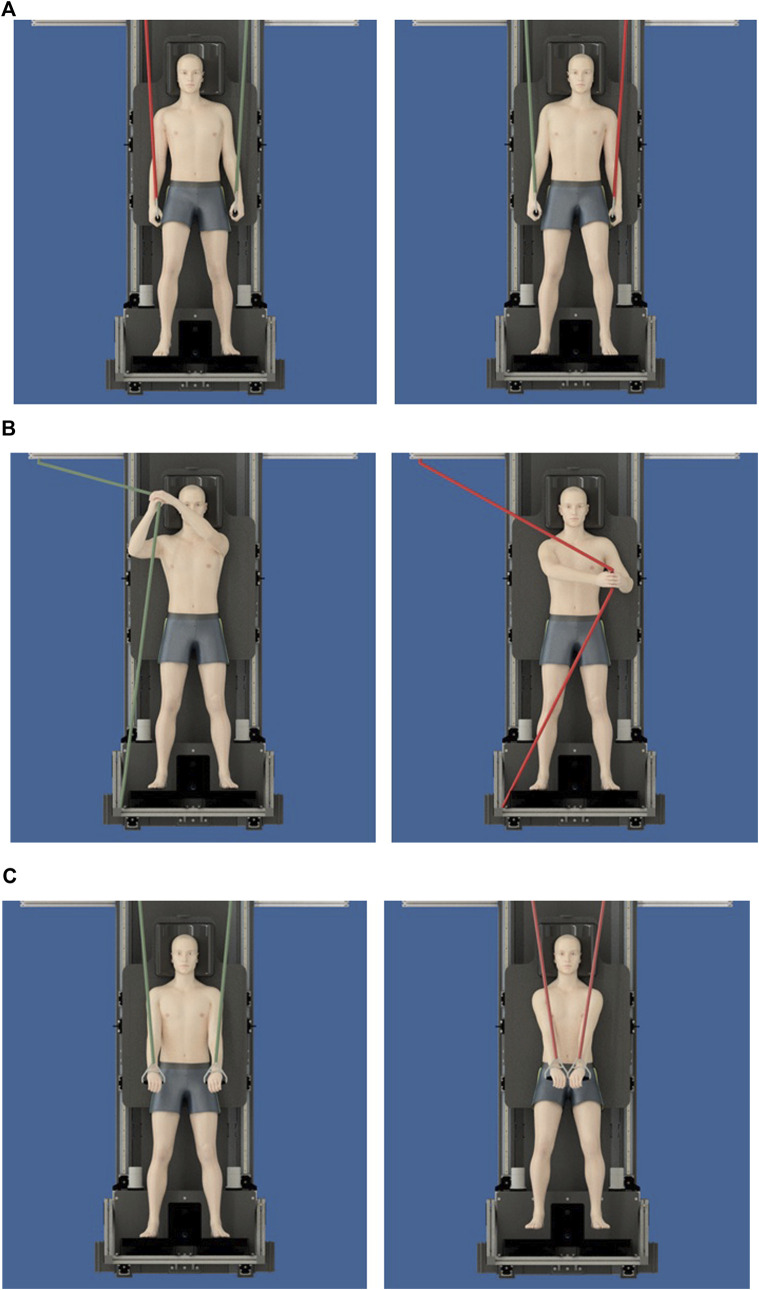
**(A)** Contralateral exercises per set were performed requiring participants to maintain trunk stability (straight hip) with the right arm holding suspension bands (red line) either with **(A)** left leg standing on the pillow or **(B)** right leg on the pillow. **(B)** Wood chopper exercises were performed on each side requiring participants to pull a rubber band (TheraBand^®^) with both arms’ diagonal from the shoulder down towards the feet while standing on pillows. **(C)** Crunch Exercises were performed by pushing down suspensions with both hands simultaneously while stabilizing the trunk and standing on pillows.

For contralateral exercise (Figure 2A) maintenance of diagonal trunk stability was required with the right arm holding suspension (TRX^®,^ United States) and the left leg standing on the pillow, or vice versa. Wood chopper exercise (Figure 2B) consisted of pulling a rubber band (TheraBand^®^, United States) with both arms diagonally from the shoulder down towards the feet while standing on pillows. Crunch exercises (Figure 2C) were performed by pushing down suspension bands (TRX^®^, San Francisco, United States) simultaneously with both hands while holding the trunk in position and standing on pillows.

### Trunk muscle surface electromyography

Bi-polar telemetric surface electrodes (Noraxon Ultium, United States) were placed and fixed with surgical tape bi-laterally on upper and lower rectus abdominis, obliquus externus abdominis, lumbar multifidi having shaved, exfoliated and cleaned the skin with alcohol. EMG signals were sampled at 2000 Hz and bandpass filtered (10–500 Hz). Start and end of each exercise bout indicated by the onset and offset of EMG activity were marked and root mean square (RMS) filter (100 ms window) applied. Prior to each experiment day, participants performed 3 s maximum voluntary contractions (MVC) of each muscle group (Konrad, 2005) with a rest interval of at least 1 min between maximal efforts. Verbal encouragement was given. The recorded MVC were used to normalize subsequent EMG signals (%MVC) averaged (left and right) per muscle.

### Heart rate and blood pressure monitoring

Heart rate was continuously recorded via a five-lead electrocardiogram to facilitate concurrent reporting with periodic brachial blood pressure measurements (Philips IntelliVue^®^ MP2, Eindhoven, Netherlands). On the centrifuge, blood pressure was recorded at BASELINE, after 1 g at CoM was reached (PRE-EXERCISE), within 60 s following each exercise (BREAK 1, BREAK 2, BREAK 3) and after completion of exercises (POST).

### Questionnaires

To assess susceptibility to motion sickness, participants completed a short-form motion sickness susceptibility questionnaire (Golding, 2006) at BASELINE. Furthermore, before (BASELINE) and after (POST) exercises, participants completed more detailed questionnaires including Motion Sickness Assessment Questionnaire (MSAQ), Positive and Negative Affect Schedule (PANAS), and Epworth Sleepiness Scale (ESS) questionnaires. MSAQ was employed to determine (1–9 max) various dimensions (e.g. gastrointestinal) of motion sickness (Gianaros et al., 2001). PANAS was used to assess the effect of centrifugation upon mood based on a Likert scale from 1 “not at all” to 5 “very much” (Watson and Clark, 1988). Induced drowsiness was assessed with the ESS (rating from 0 (non-) to 3 “high chance of dozing” in eight contexts) (Graybiel and Knepton, 1976).

Subjective motion sickness ratings (MS: 0 = “I am feeling fine” to 20 = “I am about to vomit”) (Young et al., 2003), perceived exertion (RPE: 6 = “No exertion at all” to 20 = “Maximal exertion”) (Borg, 1998) in addition to body control using a modified Cooper-Harper body control scale (1 = “not limited” to 10 = “body control lost”) (Cooper and Harper RJMF, 1969) were administered at BASELINE, PRE-EXERCISE, at BREAK 1, BREAK 2, BREAK three and POST.

### Statistical analysis

Linear mixed models (Satterthwaite method) were used to determine if there was an effect of condition (UPRIGHT, CLOCKWISE and COUNTER-CLOCKWISE), exercise (Contralateral, Wood Chopper and Crunch) and muscle activity (Ext. oblique, Mulitfidi, Upper Rectus Abdominis, Lower Rectus Abdominis).

We compared the effect of exercises and condition upon heart rate, blood pressure and subjective ratings (condition x time). Furthermore, we compared the effect of standing upright in 1 g vs. passive centrifugation with 1 g at the CoM in supine position (PRE-EXERCISE) upon heart rate, blood pressure and subjective ratings to evaluate the effect of solely centrifugation.

All data was normally distributed (Shapiro Wilk’s test). Data are presented as mean ± standard deviation of the mean (SD). All statistical tests were conducted using R (version 4.1.2) with *p* < 0.05 assumed as being statistically significant.

## Results

To generate 1 g at the participant´s CoM the centrifuge spin rate was 18.56 ± 0.2 rpm with a radius of 98.3 ± 5.6 cm. No exercise bouts were aborted in any condition and no adverse medical events were reported.

### Muscle activity

**FIGURE 3:**
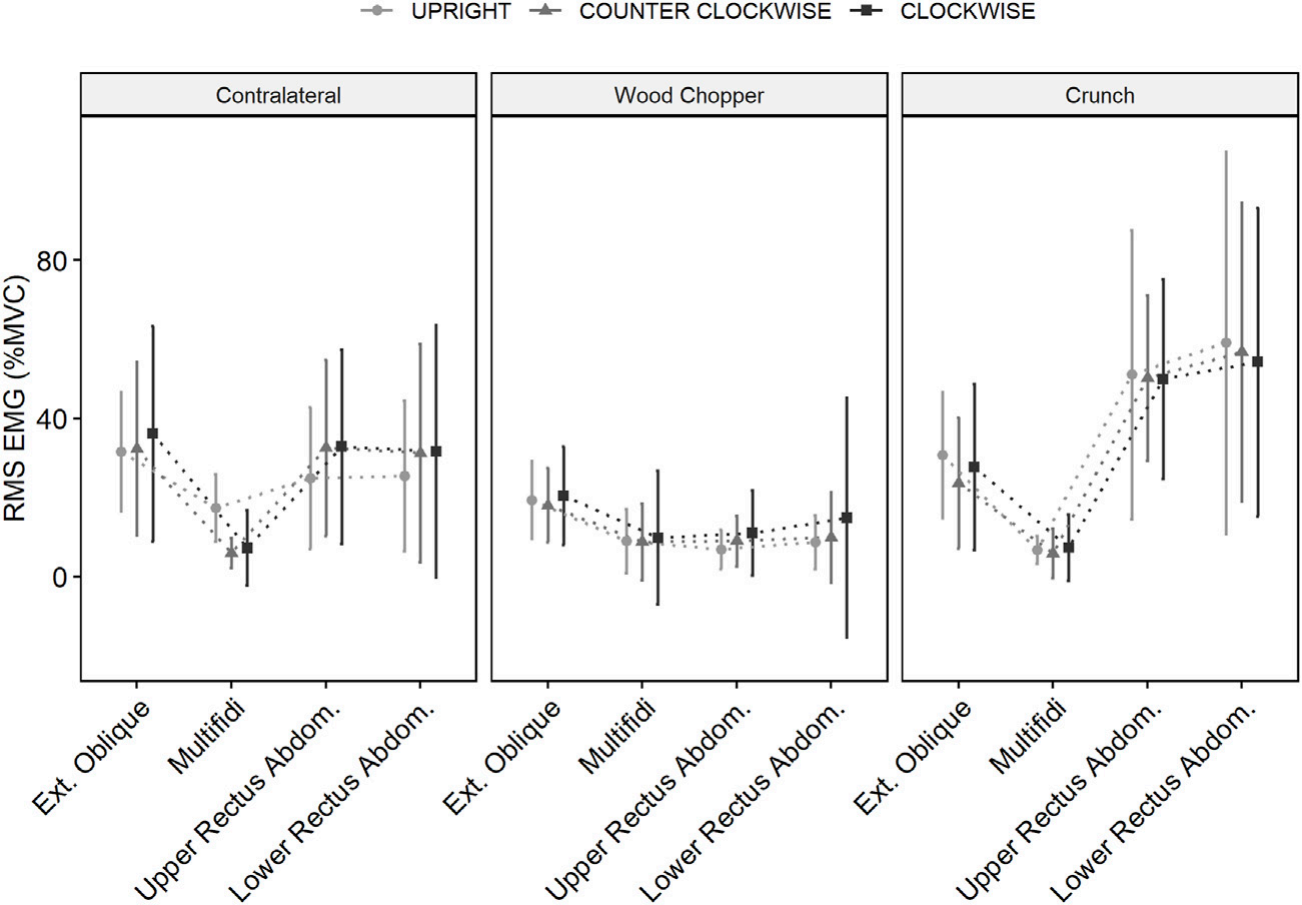
Root mean square of muscle activation (%MVC) for, External Oblique, Multifidi, Upper and Lower Rectus Abdominis during exercises (Contralateral, Wood Chopper and Crunch), when standing upright (UPRIGHT) and during short arm human centrifugation (SAHC) when rotated clockwise (CLOCKWISE and counter-clockwise (COUNTER-CLOCKWISE) when supine with 1 g at the participant’s center of mass. Data as mean (±SD).

Mean muscle activity (%RMS) during wood chopper and crunch exercise showed no significant differences whether performed during centrifugation or standing upright. However, we found a significant 3-way interaction (condition x exercise x muscle) with F = 2.4421, *p* = 0.003, df = 12 for contralateral exercise. In particular, differences were observed for multifidi muscle activity during contralateral exercise between the conditions with higher values in UPRIGHT compared to CLOCKWISE (*p* = 0.003) and COUNTER-CLOCKWISE (*p* < 0.001) (Figure 3).

Muscle activity of upper and lower rectus abdominis were significantly affected by condition during contralateral exercise, with higher activation observed during both CLOCKWISE (*p* = 0.023) and COUNTER-CLOCKWISE (*p* < 0.001) centrifugation compared to UPRIGHT exercise.

### Cardiovascular response

**FIGURE 4:**
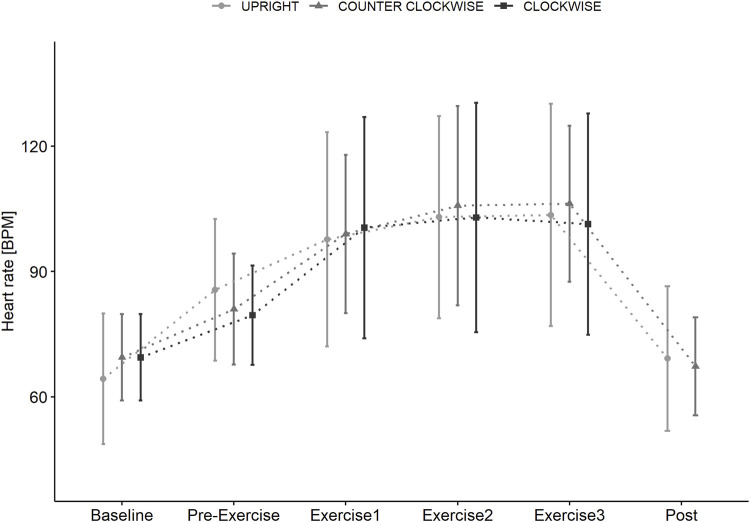
Heart rate (bpm) in supine position (BASELINE) prior to standing (PRE-EXERCISE) in the UPRIGHT condition, and when passively rotated (PRE-EXERCISE) clockwise (CLOCKWISE) and counter-clockwise (COUNTER-CLOCKWISE) with 1 g at the participants center of mass in addition to directly following Contra-lateral, Wood Chopper and Crunch exercise (BREAK 1, BREAK 2, BREAK 3) and upon completion (POST) of each condition. Data as mean (±SD).

Heart rate (Figure 4) was significantly changed over time (F = 52.8965, *p* < 0.001, dfs = 5) but not by condition (F = 0.0671, *p* = 0.935, dfs = 2). Compared to supine resting (BASELINE), heart rate was significantly increased when standing upright in 1 g vs. passive centrifugation with 1 g at the CoM in supine position (PRE-EXERCISE: *p* < 0.001) and during exercises (BREAK 1: *p* < 0.001, BREAK 2: *p* < 0.001, BREAK 3: *p* < 0.001) but returned to baseline values after completion of exercises (POST: *p* = 0.805).

**FIGURE 5:**
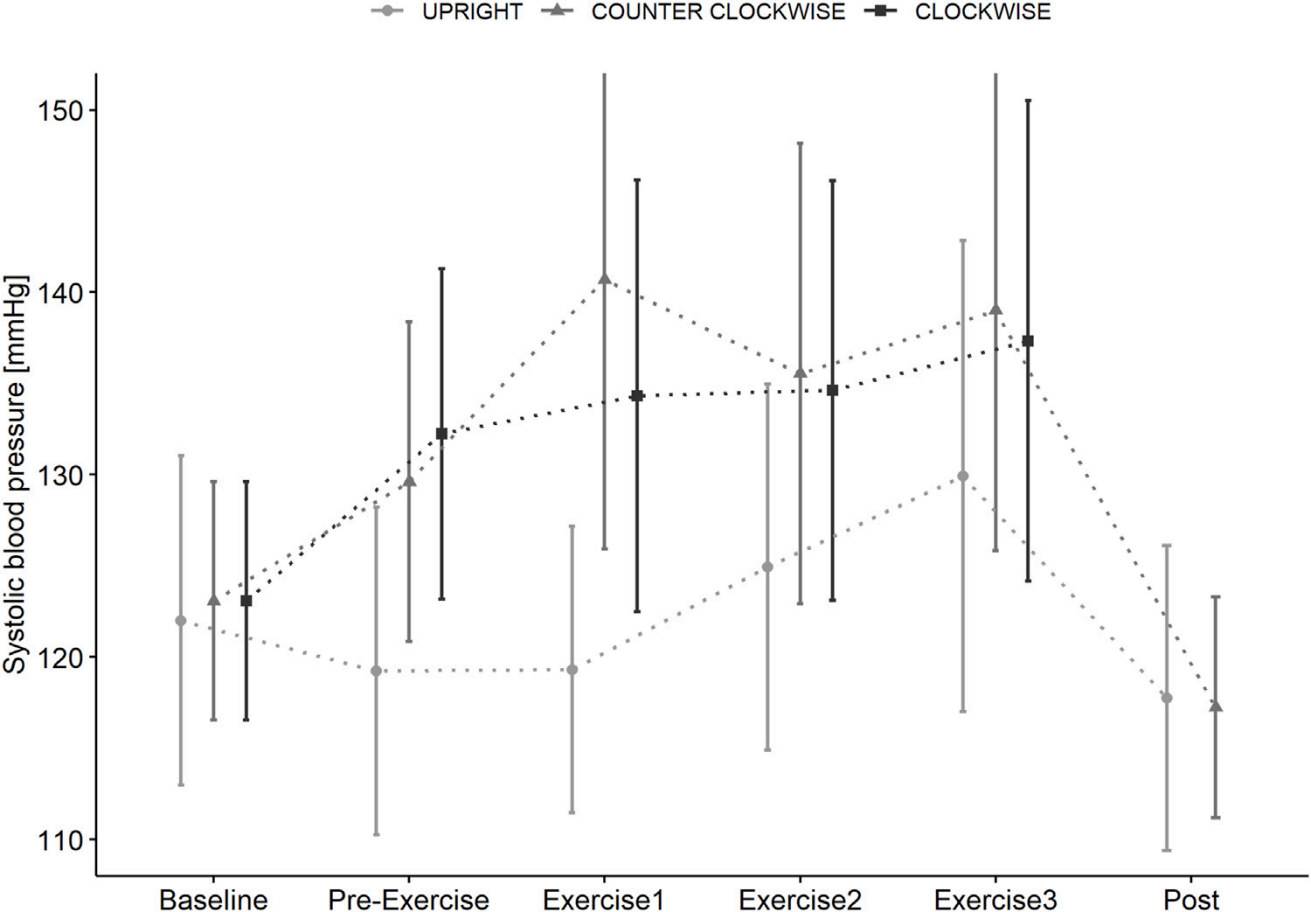
Systolic blood pressure measured in supine position (BASELINE) prior to standing (PRE-EXERCISE) in the UPRIGHT condition, and when passively rotated (PRE-EXERCISE) clockwise (CLOCKWISE) and counter-clockwise (COUNTER-CLOCKWISE) with 1 g at the participants center of mass in addition to directly following Contra-lateral, Wood Chopper and Crunch exercise (BREAK 1, BREAK 2, BREAK 3) and upon completion (POST) of each condition. Data as mean (±SD).

Systolic blood pressure (Figure 5) showed a significant interaction effect (time x condition, *p* < 0.001) with a lower and more delayed increase during UPRIGHT (PRE-EXERCISE: ns, BREAK1: ns, BREAK 2: ns, BREAK 3: *p* = 0.007) compared to CLOCKWISE (PRE-EXERCISE: *p* = 0.016, BREAK 1: *p* = 0.005, BREAK 2: ns, BREAK 3: ns) and COUNTER-CLOCKWISE (PRE-EXERCISE: *p* = 0.016, BREAK 1: *p* < 0.001, BREAK 2: *p* = 0.014, BREAK 3: *p* = 0.033).

**FIGURE 6:**
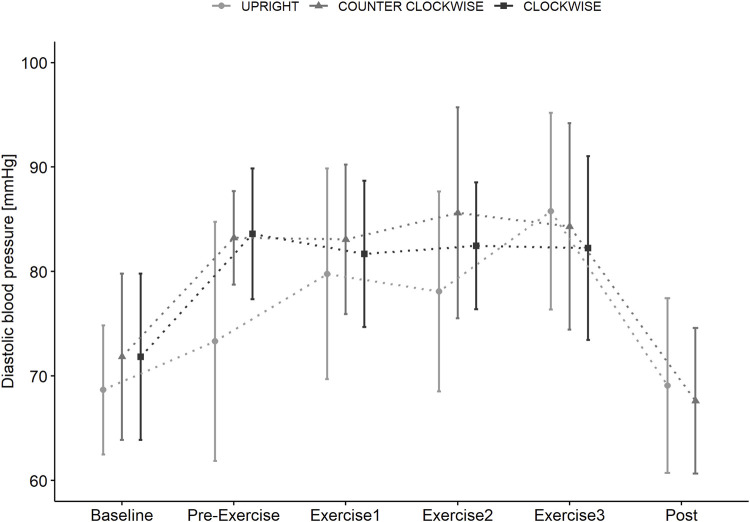
Diastolic blood pressure measured in supine position (BASELINE) prior to standing (PRE-EXERCISE) in the UPRIGHT condition, and when rotating passively (PRE-EXERCISE) clockwise (CLOCKWISE) and counter-clockwise (COUNTER-CLOCKWISE) with 1 g at the participants center of mass in addition to directly following Contra-lateral, Wood Chopper and Crunch exercise (BREAK 1, BREAK 2, BREAK 3) and upon completion (POST) of each condition. Data as mean (±SD).

Diastolic blood pressure (Figure 6) was affected by time x condition (*p* = 0.018) with lower values during UPRIGHT compared to COUNTER-CLOCKWISE (PRE-EXERCISE: *p* = 0.005, BREAK 1: ns, BREAK 2: *p* = 0.026, BREAK 3: ns) but not compared to CLOCKWISE.

### Subjective ratings

MSSQ scores were low (5.4 ± 4.8) due to MSA (3.8 ± 3.7) and MSB (1.6 ± 4.8) sub-scores. No participant reported increased motion sickness prior to, or following exercise in any condition.

MS scores were low (<3) and not affected by time (F = 0.074, p 0.974, dfs = 3) (Table 1). However, we found a significant condition effect (F = 17.528, *p* < 0.001, dfs = 2) with higher scores for CLOCKWISE (*p* < 0.001) and COUNTER-CLOCKWISE (*p* < 0.001) during BASELINE, PRE-EXERCISE and EXERCISE compared to UPRIGHT.

**TABLE 1:** Motion sickness (MS), perceived exertion (RPE) and effort ratings supine (BASELINE), prior to standing (PRE-EXERCISE) in the UPRIGHT condition, and when rotating passively (PRE-EXERCISE) clockwise (CLOCKWISE) and counter-clockwise (COUNTER-CLOCKWISE) with 1 g at the participants center of mass. Further ratings were obtained directly following Contra-lateral, Wood Chopper and Crunch exercise and upon completion (POST) of each condition. Data as mean (±SD).

	UPRIGHT			CLOCKWISE			COUNTER CLOCKWISE		
	MS	RPE	Effort	MS	RPE	Effort	MS	RPE	Effort
BASELINE	1.2 ± 0.4			2.0 ± 1.5			2.2 ± 2.5		
PRE-EXERCISE	1.2 ± 0.4	6.0 ± 0.0		2.0 ± 1.5	6.4 ± 0.7		2.2 ± 2.5	6.8 ± 1.2	
Contra Lateral	1.3 ± 0.7	9.8 ± 2.6	1.8 ± 1.4	2.1 ± 1.8	9.6 ± 2.6	1.9 ± 0.8	2.2 ± 2.5	9.6 ± 3.0	1.8 ± 0.9
Trunk exercise	1.3 ± 0.6	11.2 ± 2.6	1.8 ± 1.6	2.0 ± 1.9	10.6 ± 3.3	1.7 ± 0.7	2.2 ± 2.4	10.7 ± 3.1	1.6 ± 0.5
Wood chopper	1.3 ± 0.6	8.7 ± 2.4	2.3 ± 2.2	2.0 ± 1.9	9.1 ± 2.8	2.1 ± 1.0	2.1 ± 2.5	9.4 ± 3.0	2.0 ± 1.2
POST	1.2 ± 0.6			1.8 ± 1.7			1.8 ± 1.7		

We found a significant increase of RPE scoring over time (F = 54.185, *p* < 0.001, dfs = 3) (Table 1) but no effect of condition (F = 0.169, *p* = 0.844, dfs = 2). Effort ratings showed minor changes over time (F = 4.6149, *p* = 0.01, dfs = 2) (Table 1) without differences between conditions (F = 0.6411, *p* = 0.527, dfs = 2).

## Discussion

In this pilot trial we compared trunk muscular activity (mm. ext. obliques, multifidi and mm. rectus abdominis), cardiovascular response and tolerability of trunk muscle exercises performed during clockwise and counter-clockwise SAHC with 1 g at the individual CoM compared to that generated in an upright (1 g) position. Contralateral, Wood Chopper and Crunch trunk muscle exercises with 1 g at the CoM during SAHC were feasible and well tolerated. The main finding of the study was that trunk and back muscular activity in response to trunk exercises was comparable during centrifugation to that performed during upright standard conditions. Contralateral exercises showed lower muscle activation in the back but increased in straight abdominal muscles during centrifugation compared to upright. No significant differences pre and post exercise in MSAQ, PANAS or ESS in any conditions were found (see Table 2). No exercises were aborted in any condition either due to excessive physiological load or motion sickness.

**TABLE 2:** Motion Sickness (MSAQ), Positive and Negative Affect Schedule (PANAS), and Epworth Sleepiness Scale (ESS) scores prior to (BASELINE) and following the upright (UPRIGHT) and short arm human centrifugation conditions when rotated both clockwise (CLOCKWISE) and counter-clockwise (COUNTER-CLOCKWISE) with 1 g at the participants center of mass when supine. Data as mean (±SD).

	UPRIGHT		SAHC	
	Pre	Post	Pre	Post
MSAQ	14.5 ± 2.1	12.6 ± 2.1	12.4 ± 1.8	15.8 ± 5.4
ESS	14.8 ± 3.2	14.3 ± 3.6	15.0 ± 3.1	15.5 ± 3.0
PANAS (Positive Affect)	30.3 ± 6.0	30.1 ± 5.5	32.9 ± 5.8	31.5 ± 8.5
PANAS (Negative Affect)	13.5 ± 1.5	12.7 ± 1.1	14.6 ± 2.7	13.3 ± 0.8

Trunk muscle atrophy and reduced muscle tone (McNamara et al., 2019) may contribute to inter-vertebral disc (IVD) pathology and contributes to an apparent increased risk of IVD herniation post-flight (Johnston et al., 2010). Moderate axial loading via elasticated body suits has been suggested to promote core stability (Rathinam et al., 2013) with some developed for μg environments (Waldie and Newman, 2011) demonstrated to be compatible with aerobic (Attias et al., 2017) and resistive (Carvil et al., 2017) exercise on Earth, and on the ISS (Stabler et al., 2017). However, whilst these suits may promote trunk muscle activation during exercise, such an approach is unlikely to mitigate multi-organ deconditioning.

Recent findings suggest that passive exposure to intermittent centrifugation during 60 days head down tilt bed rest do not provide protective effects to maintain control and coordination of superficial and deep lumbar spinal muscles during anticipatory adjustments to quick arm movements (De Martino et al., 2021). In the present study target muscle activation during exercises were comparable when conducted in supine position while rotating on the centrifuge with 1 g at CoM and standing upright in 1 g condition. However, for contralateral exercise, multifidi muscle activity was significantly lower when performed on the SAHC whereas activity of upper and lower rectus abdominis were increased. No differences were observed during wood chopper and crunch exercises. Thus, trunk muscle activity was broadly similar between the two positions, with no evidence of exaggerated or potentially inappropriate activity. Interestingly, few differences were observed between SAHC rotation direction suggesting that participants were able to adapt effectively. Centrifugation without exercising elicit only minor muscular activity in leg muscles an no relevant activity in the targeted muscles.

In fact, in both upright and SAHC conditions muscular activation levels were low in comparison of the 40–60% considered to be ergogenic for abdominal musculature (Konrad, 2005). Activation of the multifidi in particular was low (below 20% MVC) in all conditions, however this is reported to be challenging to activate with exercise (Cao et al., 2005; Holguin et al., 2009), which may account for the de-conditioning observed in bedrest (Belavý et al., 2011) and spaceflight (Johnston et al., 2010; Burkhart et al., 2019), which in astronauts may underlie lumbar lordosis (Bailey et al., 2018) and vertebral column dysfunction (Lazzari et al., 2021) including IVD pathology and back pain (Pool-Goudzwaard et al., 2015). However, on Earth, a classic exercise for abdominal muscles, the static curl-up recruits approx. 80% MVC whereas back extension exercises for the erector spinae elicit approx. 63% MVC contractions (Oliva-Lozano and Muyor, 2020) Thus, whilst the muscle recruitment in our exercises appears to be moderate, the failure to observe functionally significant different with those performed upright suggests that more complicated, and or aggressive trunk muscle exercises should be evaluated acutely during SAHC. Should they prove to be feasible, tolerable and induce trunk muscle activation likely to be ergogenic then a AG-training study employing such during long duration head down tilt may be warranted to see if they plug a key operational countermeasure gap (Green and Scott, 2017) given that daily passive AG at 1 g at CoM is well tolerated during head down tilt bed rest (Frett et al., 2020a) but provides a relatively low physiological load (Kramer et al., 2020a), and appears insufficient to ameliorate multi-system bed rest-induced deconditioning (Attias et al., 2020; Martino et al., 2020; Hoffmann et al., 2021).

The transition from supine to 1 g at the CoM induced broadly similar increase in heart rate compared to standing. However, passive centrifugation showed a greater rise of systolic and diastolic blood pressure in both rotational directions indicating either a greater influence of fluid shift or stress. Trunk muscle exercise further increased heart rate and systolic blood pressure, with blood pressures being lower in the upright condition. However, differences were relatively minor and not indicative of haemodynamic challenge (Laing et al., 2020). This is perhaps unsurprisingly given the relatively low muscle mass and magnitude of muscle activation.

Ratings of perceived exertion and motion sickness ratings were moderate even in our naïve subjects, and comparable with terrestrial exercise (Scherr et al., 2013; Kim and Park, 2018) with no participant reporting significant motion sickness in any condition. This is consistent with our observation that subjects were able to perform the trunk exercises without moving their head, and recent findings demonstrating that when the g load is moderate (i.e. 1 g at CoM) and exercise-related head and body motion is congruent, movement during SAHC is well tolerated (Piotrowski et al., 2018; Frett et al., 2020b; Kramer et al., 2020b). Body control remained good in all conditions, with no effect of exercise or condition on MSAQ, PANAS and ESS scores. Whilst higher g-levels may have resulted in greater trunk muscle activation they increase the risk of disorientation and/or motion sickness (Bertolini and Straumann, 2016).

As this was a pilot study we were not prescriptive with exercise instructions. However, given the low level and range of trunk muscle recruitment an extended period of familiarization to the exercises during SAHC may be advantageous.

Our study has some limitations. Safety regulations required a solid back plate during centrifugation, limiting participant´s mobility compared to when upright control condition. This may explain the reduction in multifidi activation during lateral stabilization exercises. Development of a full-body suspension system to facilitate 3D participants mobility during SAHC is warranted. Additionally, we assume biomechanical differences due to a body position that is not aligned with the resulting vector while spinning. As the used centrifuge setup has no swing-out capability, participants experience a vector angle while spinning at +1Gz at CoM that is 45° different to the body longitudinal axis compared to standing upright in Earth´s gravity. Future ground based SAHC training studies should carefully consider a correct inclination of participants align with the resulting force vectors. Given the small sample size, we cannot differentiate for gender specific effects.

We used surface EMG to ensure natural movement patterns that are not compromised by application of intramuscular wire electrodes. As trunk muscles are multilayered and have different fiber orientations, the intensity and selectivity of the recorded signals may be compromised by using surface EMG instead of intramuscular wire electrodes due to cross-talk from adjacent muscles and a low signal-to-noise ratio.

In conclusion, this study demonstrates that Contralateral, Wood Chopper and Crunch trunk muscle exercises with 1 g at the CoM during SAHC were feasible, and well tolerated. In general, targeted trunk muscular activity was comparable to that performed during upright 1 g conditions. Heart rate responses were similar whilst blood pressure was higher during SAHC, but not indicative of excessive physiological load.

As the optimal g-level to maintain crew´s fitness during spaceflight is still unclear, we used a g-level relatively similar to terrestrial gravity in order to investigate the effects of trunk muscular exercises during centrifugation. Thus, trunk muscle exercise is a candidate for SAHC-based training on Earth, and in space. However, further optimization is required prior to performance of a training study for individuals with trunk muscle atrophy/dysfunction and/or long-term Head Down Tilt Bed Rest - an analogue of microgravity.

## Data Availability

The raw data supporting the conclusion of this article will be made available by the authors, without undue reservation.
